# Congenital Contractures and Fractures: A Variant of Bruck Syndrome Type 2

**DOI:** 10.7759/cureus.61991

**Published:** 2024-06-09

**Authors:** Pallavi Yelkur, Syed Mohammed, Kishore Narayan

**Affiliations:** 1 Pediatrics, Saveetha Medical College and Hospital, Saveetha Institute of Medical and Technical Sciences (SIMATS), Saveetha University, Chennai, IND

**Keywords:** joint contractures, plod2 gene mutation, arthrogryposis multiplex congenita, osteogenesis imperfecta, bruck syndrome

## Abstract

Bruck syndrome, an exceptionally rare autosomal recessive disorder, manifests as bone fragility and congenital joint contractures. This syndrome is recognized as a fusion of arthrogryposis multiplex congenita and osteogenesis imperfecta and is categorized into Types 1 and 2. Bruck syndrome Type 2 stems from a homozygous mutation in the PLOD2 gene and exhibits characteristics such as osteopenia, congenital contractures with pterygia, femoral bowing, club feet, postnatal shorty stature, severe limb deformity, and progressive scoliosis.

In this report, we describe the case of an infant presenting with multiple joint contractures of the distal extremities, bilateral talipes equinovarus deformity, and a history of a right femur fracture at birth, managed through closed reduction and plaster of Paris. The current treatment regimen includes physiotherapy, wrist splinting for wrist extension and thumb abduction, and serial casting of both lower limbs.

## Introduction

Bruck syndrome is an extremely rare congenital disorder with an incidence of less than one in a million. It is classified into two types. Type 1 is characterized by the presence of osteogenesis imperfecta and pterygia (webbing of the skin) without contractures, while Type 2, also known as Bruck Syndrome with congenital contractures, includes osteogenesis imperfecta with joint contractures, as seen in arthrogryposis. Individuals with Bruck syndrome Type 2 typically present with multiple fractures of varying severity, joint contractures, and skeletal abnormalities. The joint contractures can affect the range of motion in the limbs and lead to muscle weakness. Fractures may occur with minimal trauma or even spontaneously due to the fragility of the bones. Bruck syndrome Type 1 results from a homozygous mutation in the FKBP10 gene, while Type 2 is caused by a homozygous mutation in the PLOD2 gene. 

Arthrogryposis multiplex congenita encompasses a range of symptoms rather than a distinct diagnosis and is associated with various identifiable syndromes. Arthrogryposis multiplex congenita commonly presents as congenital joint contractures. The diagnosis of Bruck syndrome occurs when congenital joint contractures are concurrent with bone fragility and fractures, classified as an autosomal recessive form of osteogenesis imperfecta [1]. Until 2015, literature had only documented this rare disorder in 27 patients [2]. In 1989, Viljoen et al. reported five children with congenital joint contractures and fractures [3], acknowledging similarities described by Dr. Alfred Bruck in 1897, thus coining the term “Bruck syndrome.” The genotype and phenotype of Bruck syndrome exhibit variability. Despite unaffected genes encoding collagen 1 chains, biochemical evidence suggests a deficiency in hydroxylation of lysine residues in collagen 1 telopeptides [4]. Herein, we present a case of a neonate displaying congenital joint contractures coupled with bone fragility and fractures, fulfilling the clinical criteria for Bruck syndrome [2]. 

## Case presentation

An infant, born from parents in a second-degree consanguineous marriage, presented with an inability to flex and extend the upper and lower limbs, along with bilateral talipes equinovarus deformity. Weighing 2.5 kg at birth, he was delivered by cesarean section at a government hospital and required Neonatal Intensive Care Unit admission for three days due to perinatal asphyxia. At birth, the child displayed multiple flexion contractures at the elbows, wrists, and knees, in addition to bilateral talipes equinovarus deformity. Notably, he also had a history of a right femur fracture at birth, treated by closed reduction and Plaster of Paris. Antenatally, there were no exposures to drugs or radiation, and the pregnancy was uneventful. He is the second child in the family, with no history of abortion, stillbirth, or sibling death, and no known bone or connective tissue disorders in the family history. The child has received immunizations, and his older brother is healthy and thriving in his studies.

**Figure 1:**
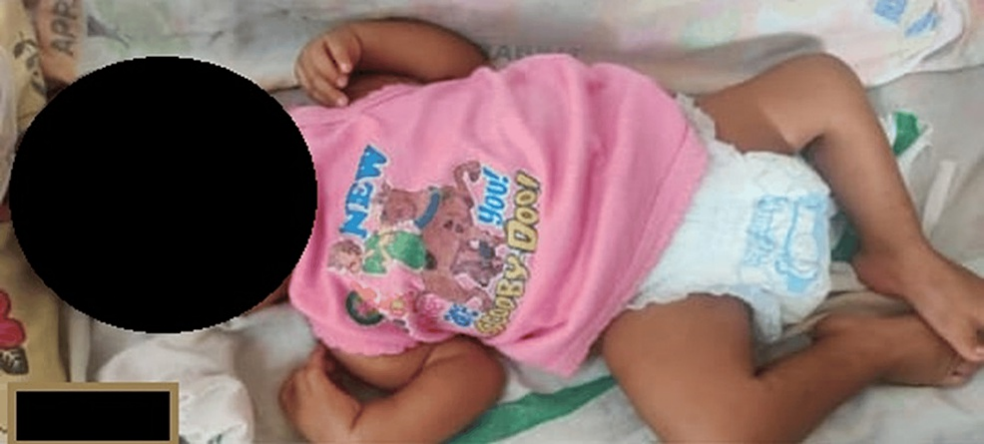
Infant with multiple flexion contractures of elbows, wrist, knees, and bilateral congenital talipes equinovarus deformity

During examination, the infant remained active with a weight of four kilograms, a length of 53 centimeters, and a head circumference of 38 centimeters, all falling below -3 Z-score according to WHO growth charts. Contractures were observed in the distal parts of all four limbs, while the anterior fontanelle was normal, and the posterior fontanelle was closed. Vitals and perfusion were within normal limits, and no visible facial dysmorphism was noted. Confirmation of normal sclera and eyes was later provided by an ophthalmologist. Examination of joints and limbs revealed typical flexion deformities in the distal parts of all four extremities, particularly pronounced in both ankle joints (Figure 1). 

**Figure 2:**
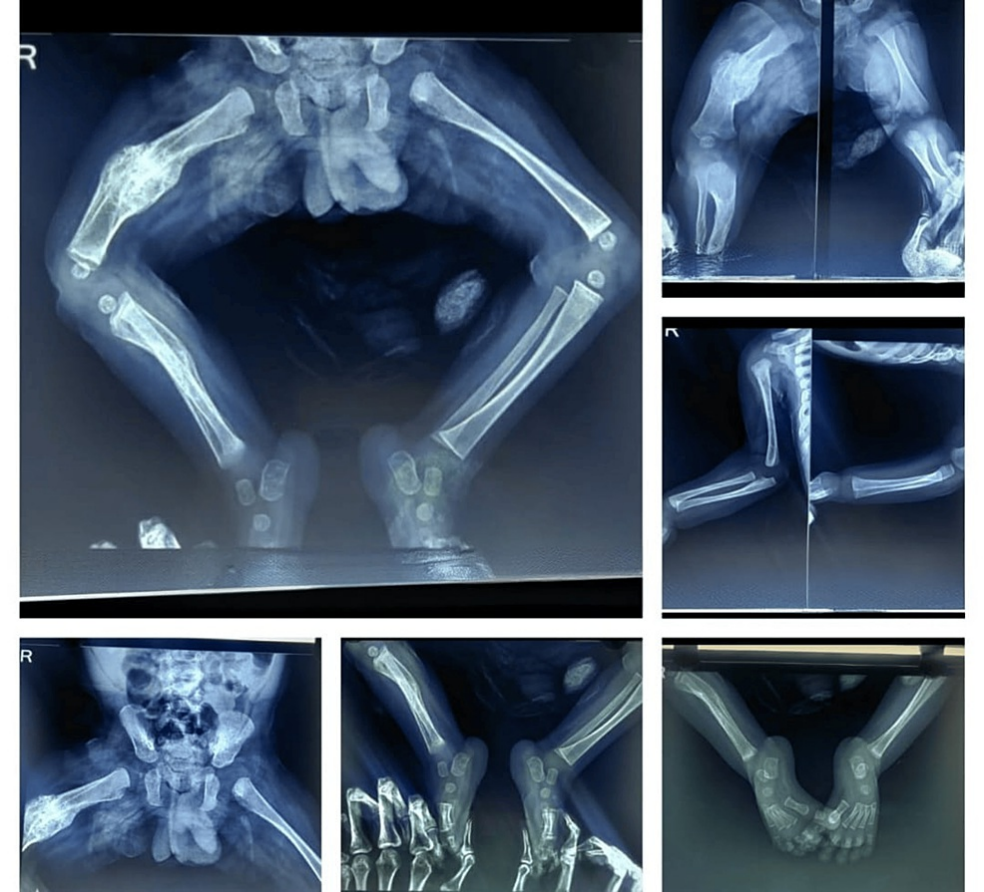
X-ray of the right femur with callus formation

Investigations revealed a normal complete blood count, along with within-normal-limits levels of calcium, serum magnesium, alkaline phosphatase, vitamin D3, parathyroid hormone, and creatinine phosphokinase. Thyroid profile results were also normal. X-rays showed a fracture of the right femur shaft with visible callus formation, while no other fractures were detected (Figure 2).

The infant's clinical presentation, including contractures in all distal limbs and a femur fracture, aligned with characteristics of arthrogryposis multiplex congenita. Genetic testing, specifically clinical exome sequencing, identified a mutation in the PLOD2 gene, confirming a diagnosis of Bruck syndrome Type 2 (Table 1). Laboratory analyses and imaging further supported the presence of bone fragility and joint contractures consistent with this diagnosis. 

**Table 1:** Clinical exome sequencing showing PLOD2 gene mutation

Variant of Uncertain Significance Related to the Given Phenotype Was Detected						
Gene (Transcript)	Location	Variant	Zygosity	Disease (OMIM)	Inheritance	Classification
PLOD2 (-) (ENST00000282903.10)	Exon 5	c.506T>C (p.Phe169Ser)	Homozygous	Bruck Syndrome-2 (OMIM#609220)	Autosomal recessive	Uncertain Significance (PM2,PP3)

## Discussion

Bruck syndrome is characterized by congenital joint contractures, pterygia, early-onset fractures, severe limb deformities, and progressive scoliosis [5]. While it is classified into two types, both share common phenotypic traits [6]. Bruck syndrome Type 2 arises from a homozygous mutation in the PLOD2 gene on chromosome 3q24, while an insertion/deletion mutation in the FKBP10 gene can also lead to the syndrome [7,8]. Features such as short stature and progressive kyphoscoliosis are often linked with Bruck syndrome Type 2 [9]. 

Bruck syndrome follows an autosomal recessive inheritance pattern, as evidenced by similar clinical features in both children despite no familial history [10]. While consanguinity may be absent in some families, it does not rule out inherited genetic mutations [10]. In areas without molecular diagnostic facilities, clinical assessment remains pivotal for understanding the underlying abnormalities. Apart from fractures and joint contractures, common features include short stature, Wormian bones, clubfoot, and kyphoscoliosis. Typically, individuals exhibit white sclera, normal hearing, and vision, though additional dysmorphic features have been noted [11-13].

Clinical outcomes vary widely, with some cases resulting in early mortality while others survive into adulthood [14-16]. The molecular defect in Bruck syndrome involves a deficiency of bone-specific telopeptide lysyl-hydroxylase, leading to abnormal collagen crosslinking [17]. This results in bone fragility, fractures, and contractures, with specific bone structure abnormalities observed on electron microscopy [18]. Differential diagnosis with osteogenesis imperfecta is imperative, necessitating early radiological imaging to prevent further fractures [14].

Bisphosphonate infusions and vitamin supplementation are the mainstays of pharmacological management, similar to osteogenesis imperfecta treatment [19]. However, prognosis is generally poorer due to joint contractures, necessitating early orthopedic intervention for rehabilitation [20]. Antenatal ultrasound can detect severe forms of Bruck syndrome, enabling genetic counseling and pregnancy termination options [21].

## Conclusions

Bruck syndrome, particularly Type 2, is a rare genetic disorder presenting with osteogenesis imperfecta and arthrogryposis multiplex congenita. Early recognition and precise diagnosis are essential for tailored orthopedic care and genetic counseling. While no cure exists, interventions like orthopedic therapy improve patient's quality of life. Research into genetic mechanisms and treatments is ongoing. Prenatal diagnosis allows for informed decisions and proactive management. Early detection remains crucial in optimizing outcomes for Bruck syndrome patients, highlighting the need for prompt intervention.
